# An 85K SNP Array Uncovers Inbreeding and Cryptic Relatedness in an Antarctic Fur Seal Breeding Colony

**DOI:** 10.1534/g3.120.401268

**Published:** 2020-06-15

**Authors:** Emily Humble, Anneke J. Paijmans, Jaume Forcada, Joseph I. Hoffman

**Affiliations:** * Department of Animal Behaviour, University of Bielefeld, Postfach 100131, 33501 Bielefeld, Germany,; ^†^ Royal (Dick) School of Veterinary Studies and the Roslin Institute, University of Edinburgh, EH25 9RG UK, and; ^‡^ British Antarctic Survey, High Cross, Madingley Road, Cambridge, CB3 OET, United Kingdom

**Keywords:** SNP chip, Affymetrix Axiom, pinniped, relatedness, runs of homozygosity

## Abstract

High density single nucleotide polymorphism (SNP) arrays allow large numbers of individuals to be rapidly and cost-effectively genotyped at large numbers of genetic markers. However, despite being widely used in studies of humans and domesticated plants and animals, SNP arrays are lacking for most wild organisms. We developed a custom 85K Affymetrix Axiom array for an intensively studied pinniped, the Antarctic fur seal (*Arctocephalus gazella*). SNPs were discovered from a combination of genomic and transcriptomic resources and filtered according to strict criteria. Out of a total of 85,359 SNPs tiled on the array, 75,601 (88.6%) successfully converted and were polymorphic in 270 animals from a breeding colony at Bird Island in South Georgia. Evidence was found for inbreeding, with three genomic inbreeding coefficients being strongly intercorrelated and the proportion of the genome in runs of homozygosity being non-zero in all individuals. Furthermore, analysis of genomic relatedness coefficients identified previously unknown first-degree relatives and multiple second-degree relatives among a sample of ostensibly unrelated individuals. Such “cryptic relatedness” within fur seal breeding colonies may increase the likelihood of consanguineous matings and could therefore have implications for understanding fitness variation and mate choice. Finally, we demonstrate the cross-amplification potential of the array in three related pinniped species. Overall, our SNP array will facilitate future studies of Antarctic fur seals and has the potential to serve as a more general resource for the wider pinniped research community.

Single nucleotide polymorphisms (SNPs) have become one of the most popular genetic markers in evolutionary and conservation biology (Morin *et al.* 2004). They are the most abundant form of genetic variation and in contrast to classical markers such as microsatellites, they can be genotyped on a very large scale (Seeb *et al.* 2011). Consequently, SNPs can provide the resolution needed to address broad-reaching questions in ecology, evolution and conservation biology with greater power than was previously possible. In particular, quantitative genetic and gene mapping studies have profited enormously from the power of these markers (Johnston *et al.* 2013; Bérénos *et al.* 2014; Barson *et al.* 2015; Gienapp *et al.* 2017).

Two of the most common approaches for genotyping SNPs in non-model organisms are genotyping by sequencing (GBS) methods such as restriction site associated DNA (RAD) sequencing (Hohenlohe *et al.* 2010; Davey *et al.* 2011) and array based methods in which panels of pre-determined polymorphisms are hybridized onto chips by companies such as Affymetrix and Illumina. GBS approaches are capable of genotyping tens of thousands of SNPs and do not necessarily require access to existing genomic resources. However, they generate large amounts of sequence data that require bioinformatic processing, which can be time-consuming and technically challenging (Shafer *et al.* 2017). An additional issue with GBS is that the depth of sequence coverage is not always high enough to call genotypes with confidence, which leads to high rates of missing data (Chattopadhyay *et al.* 2014; Huang and Knowles 2016; Benjelloun *et al.* 2019). By contrast, array-based methods are faster, require minimal technical effort, have low genotyping error rates and high call rates, and can easily be scaled up to very large numbers of individuals. SNP arrays are also flexible, with low density arrays allowing hundreds to thousands of SNPs to be genotyped and high density arrays or “SNP chips” supporting tens of thousands to millions of SNPs (Shi *et al.* 2012; von Thaden *et al.* 2020). For these and other reasons, array-based genotyping has become the method of choice for many researchers, particularly those working on long-term datasets with access to many individuals.

Until recently, the majority of array-based studies of natural populations exploited resources already developed for closely related domestic species such as the BovineSNP50 and OvineSNP50 bead chips (Pertoldi *et al.* 2009; Haynes and Latch 2012; Miller *et al.* 2012; Ogden *et al.* 2012; Johnston *et al.* 2017). However, given that cross-species polymorphism declines with increasing phylogenetic distance (Miller *et al.* 2012), custom species-specific arrays are now being developed for several wild species such as great tits (Kim *et al.* 2018), flycatchers (Kawakami *et al.* 2014a), house sparrows (Lundregan *et al.* 2018), polar bears (Malenfant *et al.* 2015) and Florida scrub jays (Chen *et al.* 2016). These resources have already provided insights into diverse topics including recombination rate variation (Kawakami *et al.* 2014b), conservation genomics (Chen *et al.* 2016) and quantitative genetics (Kim *et al.* 2018). However, high rates of failure are not uncommon with custom arrays, as considerable numbers of SNPs either fail to produce any results at all (*i.e.*, they do not “convert”) or they appear monomorphic and are consequently for most purposes uninformative. Among recent efforts to develop SNP arrays for wild organisms, the proportion of tiled SNPs converting into high quality polymorphic genotyping assays has varied from just over 50% to at most around 80% (van Bers *et al.* 2012; Hagen *et al.* 2013; Kawakami *et al.* 2014a; Malenfant *et al.* 2015; Kim *et al.* 2018). Recent studies investigating the causes of assay failure have identified poor SNP genomic context as a major factor, particularly when markers are derived from a transcriptome, and have highlighted the advantages of considering how SNP probe sequences map to a reference genome (Humble *et al.* 2016a, 2016b). Consequently, incorporating contextual information into SNP filtering pipelines has the potential to substantially improve the success rates of custom arrays.

The Antarctic fur seal (*Arctocephlaus gazella*) is a prime example of a species that would benefit from the development of a SNP array. On Bird Island in South Georgia, a breeding colony of fur seals has been intensively monitored since the 1980s and genetic, phenotypic and life-history data have been collected for around ten thousand animals. This information has provided the foundation for elucidating the species’ mating system (Hoffman *et al.* 2003, 2007), demographic history (Hoffman *et al.* 2011; Paijmans *et al.* 2020) and population status (Forcada and Hoffman 2014). For example, by combining data from nine microsatellites with multi-event mark-recapture models, Forcada and Hoffman (2014) showed that adverse climate effects have led to a 24% decline in the number of breeding females over the past three decades. Alongside this, breeding female heterozygosity has increased by around 8.5% per generation since the early 1990s (Forcada and Hoffman 2014). Together, these patterns are strongly suggestive of increasing viability selection against homozygous offspring possibly due to inbreeding depression.

To shed light on this phenomenon in fur seals as well as to improve our broader understanding of the mechanisms responsible for inbreeding depression, a shift from using small numbers of microsatellites to many thousands of SNPs is required (Kardos *et al.* 2015). High density datasets of mapped SNPs are capable of estimating inbreeding with extremely high precision because they can quantify the genome-wide contribution of runs of homozygosity (ROH), contiguous tracts of homozygous SNPs that occur when individuals inherit two identical by descent (IBD) copies of a chromosomal segment from a common ancestor (Franklin 1977). Indeed, simulation studies have shown that ROH-based measures provide more precise estimates of inbreeding than those obtained from pedigrees (Keller *et al.* 2011), which cannot capture variation among individuals due to recombination and Mendelian sampling (Hill and Weir 2011). Furthermore, the length distribution of ROH can shed light on whether inbreeding is the result of matings between relatives in recent generations or in the more distant past (Thompson 2013). This is because the length of an IBD segment is determined by the number of generations between the inbred individual and the most recent common ancestor carrying the two homologous copies of that IBD segment. For these reasons, quantifying ROH is becoming the method of choice among researchers interested in inbreeding and inbreeding depression (Kardos *et al.* 2017; Grossen *et al.* 2018; van der Valk *et al.* 2020).

As well as improving estimates of inbreeding, genome-wide marker panels have made it possible to calculate precise measures of relatedness, something that has traditionally been restricted to populations for which a pedigree is available (Santure *et al.* 2010; Huisman 2017). Understanding how animals are related is of fundamental importance to many aspects of evolutionary and conservation biology, from understanding patterns and mechanisms of mate choice (Foerster *et al.* 2006; Blyton *et al.* 2016; Tuni *et al.* 2019) to making informed pairing decisions in conservation breeding programs (Galla *et al.* 2020). As high quality, multi-generational pedigrees are not available for most wild populations, the possibility of using genomic data for deriving relatedness estimates therefore provides many additional research opportunities.

This paper describes the development of an 85K Affymetrix Axiom genotyping array for the Antarctic fur seal. As our long-term aims are to investigate the mechanism(s) behind the population decline as well as more generally to explore the genetic architecture of fitness-related traits, we developed a genome-wide panel of nuclear SNPs based on RAD sequencing data from a recent study (Humble *et al.* 2018). We additionally made use of another desirable property of SNP arrays, the possibility of incorporating candidate gene markers, by tiling over ten thousand polymorphisms from a transcriptome assembly (Humble *et al.* 2016b) together with a handful of SNPs from the major histocompatibility complex (MHC), a group of genes constituting arguably the most important component of the vertebrate immune system (Sommer 2005). Finally, we attempted to maximize the overall genotyping success of the array by subjecting all discovered SNPs to a strict prioritization scheme that incorporated multiple sources of information including the genomic context of each locus. We genotyped 288 samples, primarily from Antarctic fur seals but also including three additional pinniped species, to assess the performance of the SNP array, to quantify inbreeding and to explore patterns of relatedness among individuals.

## Materials And Methods

### Genomic SNP discovery

Genome-wide distributed nuclear SNPs were discovered using RAD sequencing as described by Humble *et al.* (2018). Briefly, tissue samples from 83 individuals were collected from the main breeding colonies across the species range: Bird Island, South Georgia (*n* = 57), Cape Shirreff in the South Shetlands (*n* = 6), Bouvetøya (*n* = 5), Îles Kerguelen (*n* = 5), Heard Island (*n* = 5) and Macquarie Island (*n* = 5). RAD libraries were prepared using a protocol with minor modifications as described in Hoffman *et al.* (2014). Read quality was assessed using FastQC v0.112 and the sequences were trimmed to 225 bp and demultiplexed using *process_radtags* in STACKS v1.41 (Catchen *et al.* 2013). To identify SNPs to include on the array, we followed GATK’s best practices workflow (Poplin *et al.* 2017) using the Antarctic fur seal genome v1.2 as a reference (Humble *et al.* 2016a). This assembly contains 6,170 scaffolds with an N50 of 6.45 Mb. The resulting SNP dataset was filtered to include only biallelic SNPs using BCFtools (Li 2011).

We then applied a set of initial quality filters using VCFtools (Danecek *et al.* 2011) to filter out low quality SNPs from our dataset. Specifically, we removed genotypes with a depth of coverage of less than five or greater than 18 to minimize spurious SNP calls due to low coverage or repetitive genomic regions. We also removed SNPs with minor allele frequencies (MAF) below 0.05 and with a genotyping rate below 60%. Next, to prepare the remaining loci for array design, we filtered out SNPs with insufficient flanking sequences by identifying and removing those with less than 35 bp on both sides. We then collated a list of probe sequences for the remaining SNPs by extracting their 35 bp flanking sequences from the Antarctic fur seal reference genome using the BEDtools command *getfasta* (Quinlan and Hall 2010).

### Transcriptomic SNP discovery and annotation

In order to allow polymorphisms residing within expressed genes to be genotyped on the array, we included SNPs discovered from the Antarctic fur seal transcriptome in our list of probe sequences. The transcriptome sequencing, assembly and SNP detection process is fully described in Humble *et al.* (2016b). In brief, testis, heart, spleen, intestine, kidney and lung samples were obtained from nine Antarctic fur seals that died of natural causes at Bird Island, South Georgia. Skin samples were additionally collected from 12 individuals from the same locality. The transcriptome was assembled in multiple iterations using 454 and Illumina sequence data from three different cDNA libraries (Hoffman 2011; Hoffman *et al.* 2013b; Humble *et al.* 2018). SNPs were then discovered using four separate genotype callers and reduced to a consensus subset that was identified by all methods. Loci with sufficient flanking sequences for probe design, and which had been assigned appropriate quality scores by Affymetrix in our previous study, were retained for array design.

Putative functions were assigned to the transcriptomic SNPs by blasting the transcripts against the SwissProt, Trembl and non-redundant BLAST databases using BLASTx v2.2.30 (Altschul *et al.* 1990) with an e-value cutoff of 1e^-04^. We then used the *total_annotation.py* script provided by the Fool’s Guide to RNAseq (De Wit *et al.* 2012) to combine all BLAST results, download Uniprot flat files and extract Gene Ontology (GO) categories. To track the number of SNPs with putative immune, growth and metabolism functions throughout the array design process, we flagged all SNPs residing within transcripts associated with the annotation terms described in Table S1.

### Pre-validated and MHC-derived SNPs

We also added to our list of probe sequences a further set of SNPs that were previously demonstrated to be polymorphic in animals from the study colony. These included 40 SNPs derived from RAD sequencing data that were validated using Sanger sequencing (Humble *et al.* 2018), 102 transcriptomic SNPs that were validated using Illumina’s GoldenGate assay (Hoffman *et al.* 2012) and 173 cross-amplified SNPs from the Illumina Canine HD BeadChip that were previously found to be polymorphic in 24 Antarctic fur seals (Hoffman *et al.* 2013a). In addition to these, we included a further six SNPs that were recently discovered from the second exon of the Antarctic fur seal MHC class II DQB locus based on Illumina MiSeq data from 82 Antarctic fur seals (Ottensmann 2018).

### SNP selection

We took our combined list of probe sequences, comprising genomic and transcriptomic SNPs together with pre-validated and MHC-derived SNPs, and evaluated their suitability for inclusion on an Affymetrix Axiom SNP genotyping array. First, we assessed the genomic context of each SNP by blasting their flanking sequences against the fur seal reference genome using BLASTn v2.2.30 with an e-value threshold of 1e^-12^. We then determined the total number of mappings and the alignment length of the top BLAST hit. Finally, all of the probe sequences were sent to Affymetrix who assigned recommendations to each SNP using an *in silico* evaluation tool. This tool considers probe sequence characteristics such as GC content and flanking sequence duplication and calculates a probability of successfully converting into a genotyping assay for each locus. We then prioritized a list of SNPs to be included on the array based on the following criteria:

- Priority one was assigned to SNPs with an Affymetrix recommendation of “recommended” in either the forward or reverse direction, that mapped uniquely and completely to the reference genome and that were neither an A/T nor a C/G SNP, as these require twice the number of probes. We also assigned priority one status to all pre-validated and MHC-derived SNPs regardless of their Affymetrix design scores.
- Priority two status was assigned to the remaining loci if they had a “neutral” recommendation by Affymetrix in either the forward or reverse direction, mapped uniquely and completely to the reference genome, were neither an A/T nor a C/G SNP and had no secondary SNPs present within the flanking sequence.
- Priority three status was assigned to any remaining RAD loci with an Affymetrix recommendation of “recommended” in either the forward or reverse direction, that mapped to no more than two different locations in the reference genome, that were neither an A/T nor a C/G SNP and had a MAF of at least 0.017 in South Georgia (equivalent to the minor allele having been found in at least two individuals in the discovery pool for this population). The latter filter was applied in order to prioritize SNPs that were polymorphic in our study population.
- Priority four status was assigned to the remaining RAD loci with an Affymetrix recommendation of “recommended” in either the forward or reverse direction, that mapped to no more than three different locations in the reference genome and that were neither an A/T nor a C/G SNP.
- Priority five status was assigned to any high quality A/T or C/G SNPs that were assigned an Affymetrix recommendation of “recommended” in either the forward or reverse direction and that mapped uniquely and completely to the reference genome.
- Priority six status was assigned to all of the remaining RAD loci with a “neutral” recommendation in either the forward or reverse strand, that mapped to no more than two different locations in the reference genome, that were neither an A/T nor a C/G SNP and that had no secondary SNPs present within the flanking sequence.
- Priority seven status was assigned to all remaining RAD SNPs with neutral recommendations for either the forward or reverse strand.

Any SNPs remaining after these prioritization steps were assigned a priority of zero and were no longer considered for array design. After determining the priority of each SNP, we then thinned the dataset so that all RAD derived SNPs with a priority greater than or equal to three were at least 1 kb from the next adjacent SNP, and all SNPs with a priority of one or two were at least 100 bp apart. Finally, we removed 289 duplicate SNPs that were discovered by more than one approach. The final set of 87,608 SNPs was submitted to Afymettrix for Axiom myDesign chip manufacture.

### Genotyping

To assess the performance of the genotyping array, a total of 288 samples on three 96 well plates were genotyped on a Gene Titan platform by the Beijing Genomics Institute (BGI). To estimate the overall genotyping error rate, a single fur seal individual was genotyped three times, once on each plate. The majority of samples (*n* = 276) were collected from Antarctic fur seals at Bird Island, South Georgia as part of a long-term monitoring study conducted by the British Antarctic Survey. These were made up of females born between 1984 and 2016 and included 53 mother-offspring pairs. Additionally, we evaluated cross-species amplification by genotyping four samples each of three pinniped species including one phocid (the gray seal, *Halichoerus grypus*) and two otariids (the Steller’s sea lion, *Eumetopias jubatus*, and the Galápagos sea lion, *Zalophus wollebaeki*). DNA was extracted using a standard phenol-chloroform protocol (Sambrook and Russell 2006) and quantified using PicoGreen on a TECAN Infinite 200 PRO plate reader. A total of 271 samples had DNA concentrations above the manufacturer’s recommendation of 50 ng/µl. The remaining 15 samples had DNA concentrations between 40 and 50 ng/µl (*n* = 7) or between 20 and 40 ng/µl (*n* = 8). These were included to evaluate how samples with suboptimal DNA concentrations would perform on the array.

The resulting genotype data were analyzed using Affymetrix Power Tools (APT) command line software. We applied two workflows to the data, the first to assess the performance of the array in the Antarctic fur seal, and the second to quantify rates of cross-species amplification. For the former, we excluded samples belonging to the three additional pinniped species so that their inclusion did not impact overall cluster quality, and then filtered out samples with dish QC scores less than 0.82 and with call rates below 97%. For the latter, we excluded samples with dish QC scores below 0.82 but did not filter on the basis of call rate in order to retain as many samples from the other pinniped species as possible.

Genotyping was conducted for both datasets using the *apt_genotype_axiom* function in APT, with quality metrics and classifications being assigned to individual SNPs using the *Ps_Metrics* and *Ps_Classification* functions respectively. We then used the *OTV_Caller* function in the SNPolisher R package to recover SNPs that were originally classified as “off-target variants”. The resulting output was then re-classified using the APT functions *Ps_Metrics* and *Ps_Classification*. To estimate the genotyping error rate, we quantified the probability at each typed locus of both alleles being IBD between replicate samples using the Z2 score output of the –genome command in PLINK v1.9 (Purcell *et al.* 2007). We estimated MAF at each typed locus using the –freq function in PLINK and excluded duplicates and pups from the calculation to avoid any potential biases resulting from pseudo-replication.

**Figure 1:**
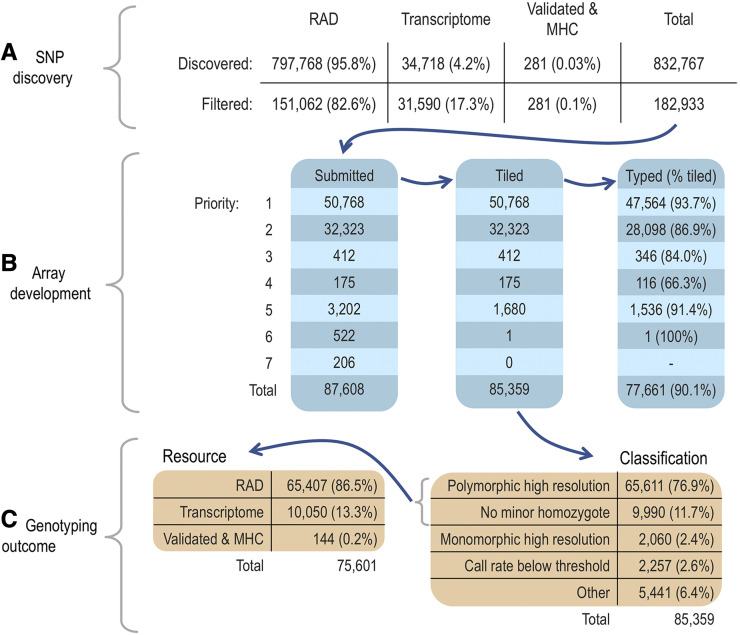
Flow diagram outlining the number of SNPs at each step of the array development pipeline. (A) Numbers of SNPs discovered, filtered and submitted for array design; (B) Numbers of submitted, tiled and genotyped SNPs in priority categories one to seven; (C) Classification outcomes of genotyped SNPs and the breakdown of resource categories for polymorphic SNPs.

We quantified the effects of our selection criteria on SNP conversion success (defined as whether a tiled SNP was successfully genotyped; Figure 1B) using a generalized linear model. The response variable SNP conversion was assigned a value of one if the SNP was successfully genotyped, or a zero if it was not successfully genotyped, and therefore we used a binomial distribution with a logit link in the model. As predictors, we fitted the following variables: Affymetrix recommendation (*affy*, 0 = neutral, 1 = recommended), pre-validated and originating from the Illumina Canine BeadChip (*canine*, 0 = false, 1 = true), transcriptomic SNP pre-validated on the GoldenGate assay (*goldengate*, 0 = false, 1 = true), RAD SNP pre-validated using Sanger sequencing (*sanger*, 0 = false, 1 = true), mapped uniquely and completely to the reference genome (*unique*, 0 = false, 1 = true) and originated from RAD loci (*rad*, 0 = false, 1 = true). For each of the predictors we reported model estimates and confidence intervals (CIs) from parametric bootstrapping on the log-odds scale. To translate log odds into probabilities, we took the inverse logit of the log odds estimate for a given combination of variables. For example, we calculated the probability of success for SNPs that were recommended by Affymetrix (*affy* = 1), mapped uniquely and completely to the reference genome (*unique* = 1) and originated from RAD loci (*rad* = 1), but were not pre-validated (*canine* = 0, *goldengate* = 0 and *sanger* = 0) as follows:

$$logit^{-1}(\beta_{0}+1\;\times \;\beta_{1}+\;0\;\times \;\beta_{2}+0\;\times \;\beta_{3}+0\;\times \;\beta_{4}+1\;\times \;\beta_{5}+\;1\;\times \;\beta_{6})$$

where $\beta_{0}$ is the estimated log-odds intercept and $\beta_{1}-\beta_{6}$ are the estimated log-odds slopes for *affy*, *canine*, *goldengate*, *sanger*, *unique* and *rad* respectively.

### Inbreeding

The genomic data were subsequently used to estimate levels of inbreeding in our study population. To ensure that sex-linked markers did not affect our estimates of inbreeding, we mapped the SNP flanking sequences to the dog genome (*Canis lupus familiaris* assembly CanFam3.1, GenBank accession number GCA_000002285.2), for which the X chromosome is well assembled, using BWA MEM with the default parameters (Li 2013). SNPs whose flanking sequence aligned to the X chromosome were identified and removed from our dataset. We also removed the duplicate individuals from our dataset, retaining the sample with the highest genotyping rate. To generate a high-quality dataset with minimal missing data, we then used PLINK to extract polymorphic SNPs and to retain loci with a genotyping rate of over 90%, MAF > 0.01 and that conformed to Hardy-Weinberg equilibrium with a *p*-value threshold of 0.001. Using the resulting dataset of 73,979 SNPs genotyped in 270 individuals, we calculated three genomic estimates of inbreeding for each individual: standardized multi-locus heterozygosity (sMLH), a measure based on the correlation of uniting gametes (${\hat{F}}_{III}$) and the proportion of the genome in ROH (*F*_ROH_). sMLH was calculated using the R package inbreedR (Stoffel *et al.* 2016) and ${\hat{F}}_{III}$was calculated using the –ibc function in GCTA (Yang *et al.* 2011).

To calculate *F*_ROH_, we first identified ROH using the –homozyg function in PLINK with a sliding window of 20 SNPs (–homozyg-window-snp 20). A window was defined as homozygous when it contained no more than one heterozygous site (–homozyg-window-het 1) and no more than five missing sites (–homozyg-window-missing 5). If at least 5% of all windows containing a given SNP were defined as homozygous, the SNP was presumed to lie within a homozygous segment (–homozyg-window-threshold 0.05). Homozygous segments were then called as ROH when they contained at least 20 SNPs (–homozyg-snp 20) and no more than one heterozygous site (–homozyg-het 1). Furthermore, to ensure that incomplete marker information did not bias ROH detection, segments were only called as ROH when they contained at least one SNP per 100 kb (–homozyg-density 100) and were at least one Mb in length (–homozyg-kb 1000). If two SNPs within an ROH segment were further than 1000 kb apart, the ROH was split into two segments (–homozyg-gap 1000). The proportion of the genome in ROH (*F*_ROH_) was then calculated as the sum of the detected ROH lengths for each individual over the total assembly length (2.3 Gb). In addition to these inbreeding estimators, we also quantified the extent of identity disequilibrium using the measure $g_{2}$ in inbreedR.

### Relatedness

Next, we used the SNP dataset to infer patterns of relatedness among the Antarctic fur seal individuals. For this analysis, we pruned the dataset of polymorphic SNPs for linkage disequilibrium using the –indep function in PLINK. We used a sliding window of 50 SNPs, a step size of 5 SNPs and removed all variants in a window above a variance inflation factor threshold of 2, corresponding to *r^2^* = 0.5. We then excluded SNPs that deviated significantly from HWE as described above. Finally, in order to retain a subset of SNPs that contained as much information as possible for inferring relationships among individuals, we filtered out loci with MAF below 0.3 and that had been called in fewer than 90% of individuals. Based on the resulting dataset of 6,575 SNPs, we quantified relatedness among all 270 individuals using three different approaches.

First, we used the program NgsRelate v2 (Korneliussen and Moltke 2015) to estimate KING-robust kinship, R0 and R1 coefficients for each pair of individuals (Waples *et al.* 2019). These statistics are based on genome-wide patterns of identity by state sharing between two individuals, where at a given SNP there are nine possible genotype combinations. R0, R1 and KING-robust kinship are each a function of different subsets of these nine values (see Figure 1 in Waples *et al.* 2019). Critically, these statistics can be calculated without allele frequency information and are robust to SNP ascertainment bias, making them ideally suited to SNP array data. Plotting R1 against R0 and KING-robust kinship should theoretically result in minimal overlap between relationship classes and therefore provides an intuitive approach for visualizing the relatedness structure of a dataset (Waples *et al.* 2019).

We next inferred relatedness categories for each pair of individuals based on theoretical expectations for different familial relationships. While Waples *et al.* (2019) derived the joint ranges of expected values for R1∼R0 and KING-robust kinship∼R1, these are restricted to a fixed data point for parent-offspring relationships (PO) and only vary on a single axis for unrelated individuals. We therefore inferred relationships using the method described in Manichaikul *et al.* (2010), for which the theoretical expectations for different familial relationships encompass a broader parameter space. This method is based on the relatedness coefficients Z0, Z1 and Z2, which reflect the proportion of the genome where a pair of individuals share zero, one or two alleles identical by descent (IBD). We therefore used the –genome function in PLINK to estimate these parameters, together with the relatedness coefficient PI_HAT, which reflects the overall proportion of the genome that is IBD. We then assigned relationship categories to each pair of individuals by comparing the estimated relatedness coefficients with the thresholds derived in Manichaikul *et al.* (2010) and provided in Table S2. This resulted in five relatedness categories: (i) parent-offspring; (ii) full-siblings; (iii) second-degree relatives such as half-siblings, avuncular relationships and grandparent-grandchild relationships; (iv) third-degree relatives such as cousins; and (v) unrelated individuals. To determine the number of difficult to call relationships, we identified pairs of individuals that were within 0.01 of the inference thresholds following Waples *et al.* (2019). Pairs of individuals that did not fall within the theoretical ranges of any category were classified as unknown.

Finally, we used the R package sequoia v2.0.7 (Huisman 2017) to assign relationship categories. We first ran an initial iteration of parentage assignment to identify potential duplicate individuals as well as loci with high error rates by setting *MaxSibIter* to zero. We then ran a second iteration of sequoia to assign siblings and second-degree relationships by setting *MaxSibIter* to 20. For both iterations, birth year information was provided using the *LifeHist* parameter. To identify likely second-degree relatives, we ran the function *GetMaybeRel*.

### Cross-species amplification potential

Finally, we investigated the cross-amplification potential of the array by quantifying the number of markers that could be successfully called in the gray seal, the Galápagos sea lion and the Steller’s sea lion using the –missing function in PLINK. We furthermore quantified the proportion of called SNPs that were polymorphic in each species.

### Data availability

The full dataset of 77,661 SNPs genotyped in 276 Antarctic fur seal samples and flanking sequences for the 85,359 tiled SNPs are available at figshare: https://doi.org/10.25387/g3.12458255. Code for the downstream genotyping analyses is available at https://github.com/elhumble/Agaz_85K_workflow_2018 Supplemental material available at figshare: https://doi.org/10.25387/g3.12458255.

## Results

### Overview

We discovered SNPs from a combination of genomic and transcriptomic resources, applied appropriate downstream filters, and then selected the most suitable loci for tiling on a custom Antarctic fur seal SNP array according to the priority scheme described in the Materials and methods. Figure 1 summarizes the design and implementation of the array including the number of SNPs retained at each step of the selection procedure and the genotyping outcomes for different types and priority categories of SNP.

### SNP discovery, filtering and array design

RAD sequencing data from 83 individuals were used to call a total of 797,768 biallelic SNPs with GATK’s best practices workflow (Humble *et al.* 2018). Downstream filtering for depth of coverage, MAF and genotyping rate resulted in a total of 151,063 SNPs, of which 151,062 had sufficient flanking sequences for probe design. A further 34,718 high quality SNPs were discovered from the Antarctic fur seal transcriptome, of which 32,727 had sufficient flanking sequences for probe design and 31,590 had appropriate Affymetrix quality scores (Humble *et al.* 2016b). Combining the RAD and transcriptomic SNPs resulted in a total of 182,652 loci. These were pooled together with 275 pre-validated SNPs and six SNPs from the MHC to produce a total of 182,933 markers to be considered for array development (Figure 1A). To select the most suitable SNPs for array design, we considered the type of SNP, genomic context, Affymetrix design score metric, pre-validation status, MAF and spacing of each locus. Based on this information, a total of 87,608 SNPs were assigned to priority categories one to seven and were sent to Affymetrix for printing. Of these, 85,359 (97%) were successfully tiled on the array, of which 59.5% belonged to the highest priority category (Figure 1B).

### Performance of the array

To evaluate the performance of the array, we genotyped a total of 274 Antarctic fur seal individuals across three microtiter plates. To provide a positive control and for genotyping error rate estimation, one of these individuals was genotyped in triplicate, once on each plate. Consequently, the total number of Antarctic fur seal samples genotyped on the array was 276. Four of these samples either failed quality control (*n* = 1) or fell below the call rate threshold of 97% (*n* = 3) and were therefore removed from the dataset. The remaining 272 samples were successfully genotyped at 77,661 SNPs, corresponding to an overall success rate of 90.1% (Figure 1B). These included 163 SNPs that were recovered after having been originally classified as “off-target variants”. The error rate determined from the individual genotyped in triplicate was low at 0.004 per locus.

To evaluate the success of our selection criteria, conversion rates (defined as the proportion of SNPs yielding high quality genotypes) were quantified separately for each priority category. SNPs assigned to priority categories one, five and six had conversion rates in excess of 90% (Figure 1B). Conversion rates were slightly lower (≥ 80%) for priority two and three SNPs, while loci assigned to priority category four had the lowest overall conversion rate of 66.3%. We explored this in more detail by modeling SNP conversion success as a function of five binary predictor variables as described in the Materials and methods (Table 1). Conversion success was higher for SNPs that were recommended by Affymetrix (β = 0.85, 95% CI = 0.80–0.89, *P* = < 2^e-16^), that mapped uniquely and completely to the genome assembly (β = 1.46, CI = 1.25–1.66, *P* = < 2^e-16^) and that originated from RAD loci (β = 0.26, CI = 0.20–0.33, *P* = 2.32^e-16^). Surprisingly, SNPs originating from the Illumina Canine HD BeadChip had a lower rate of conversion success (β = -3.19, CI = -3.52–-2.88, *P* = < 2^e-16^). There was no effect on conversion success if SNPs had been pre-validated using the GoldenGate assay (β = -0.17, CI = -0.81–0.59, *P* = 0.62) or Sanger sequencing (β = -0.09, CI = -1.11–1.34, *P* = 0.89). Overall, SNPs that were recommended by Affymetrix, that mapped uniquely and completely to the reference genome, and that originated from RAD loci had an estimated probability of conversion success of 94.1% (CI = 92.9–95.2%).

**Table 1:** Summary statistics for a binomial generalized linear model of SNP conversion success. Shown are the log-odds model estimates with standard errors and confidence intervals from parametric bootstrapping, test statistics (Z values) and *P*-values. The model had 85,352 degrees of freedom and was based on a dataset of 85,359 tiled SNPs

Term	Estimate	Std. error	95% CI	Z value	*P*
*affy*	0.85	0.02	0.80 – 0.89	34.19	< 2^e-16^
*canine*	−3.19	0.16	−3.52 – -2.88	−19.46	< 2^e-16^
*goldengate*	−0.17	0.35	−0.81 – 0.59	−0.50	0.62
*sanger*	−0.09	0.60	−1.11 – 1.34	−0.15	0.89
*unique*	1.46	0.10	1.25 – 1.66	14.22	< 2^e-16^
*rad*	0.26	0.03	0.20 – 0.33	7.92	2.32^e-15^

Overall, no relationship was found between genotyping success, expressed as the call rate per sample, and DNA concentration (Figure S1, slope = -3.4, test statistic = -1.10, *n* = 282, df = 280, *P* = 0.27). All fifteen of the samples submitted for genotyping with DNA concentrations below the manufacturer’s recommendation of 50 ng/µl had call rates above 98%, whereas the four samples that were excluded from the final dataset on the basis of suboptimal quality or call rates had DNA concentrations above 50 ng/µl.

### Levels of polymorphism

A total of 75,601 SNPs were polymorphic, equivalent to 88.6% of the tiled loci or 97.3% of the successfully converted loci (Figure 1C). The average and minimum call rates for all polymorphic SNPs were 99.6% and 93.7% respectively. The average and minimum call rates for SNPs classified as polymorphic prior to the recovery of OTVs were 99.6% and 97.04% respectively. The final dataset of polymorphic loci comprised 65,407 SNPs discovered from the RAD sequencing data, 49 SNPs that were cross-amplified from the Illumina Canine HD BeadChip and 10,142 transcriptomic SNPs, which include 92 pre-validated SNPs and three SNPs from the MHC. The loci originating from the RAD data were distributed across 835 genomic scaffolds and had a mean spacing of 35.5 kb (range = 0.02–3306.6 kb, Figure S2). The transcriptomic loci included 1,137 SNPs residing within genes with annotations relating to immunity plus 1,310 SNPs residing in genes with annotations involving metabolism and growth.

**Figure 2:**
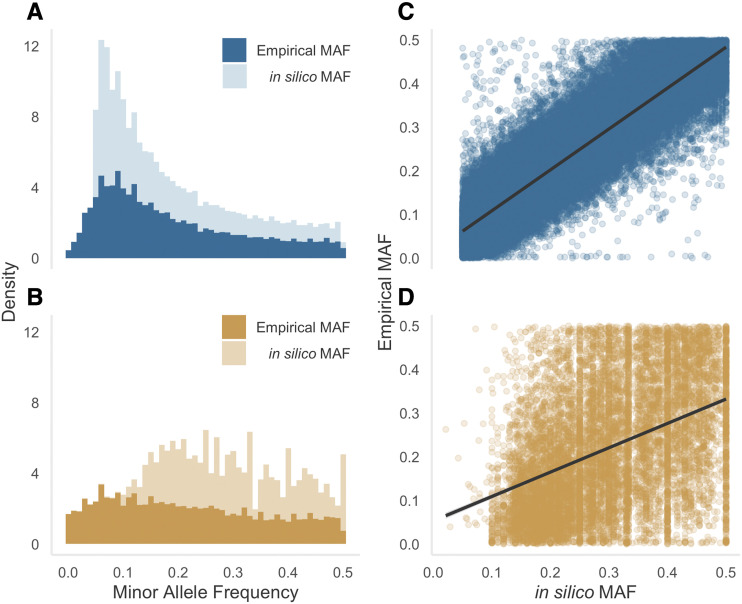
Inferred levels of SNP variability in Antarctic fur seals genotyped at 75,601 loci. Minor allele frequency (MAF) distributions of (A) RAD; and (B) transcriptomic SNPs. Panels on the right-hand side show the strength of association between empirical and *in silico* MAF for (C) RAD and (D) transcriptomic SNPs.

Focusing on the polymorphic loci, we investigated patterns of genetic variability by deriving MAF distributions separately for the RAD and transcriptomic SNPs. We also examined the correspondence between variability inferred from animals genotyped on the array (“empirical MAF”) and variability inferred from the original genomic and transcriptomic resources (“*in silico* MAF”). Empirical MAF was right skewed among the RAD SNPs (Figure 2A, mean = 0.19 +/− 0.13 SD) whereas the transcriptomic SNPs were more evenly distributed across the site frequency spectrum (SFS) (Figure 2B, mean = 0.22 +/− 0.14 SD). The empirical MAF distributions of both classes of marker extended down to zero (Figure 2A–B), whereas the corresponding *in silico* values were truncated to 0.05 due to filters applied during the SNP discovery process. A strong positive association was found between empirical and *in silico* MAF for the RAD SNPs (Figure 2C, correlation coefficient = 0.90) but this was somewhat weaker for the transcriptomic SNPs (Figure 2D, correlation coefficient = 0.43).

### Inbreeding

**Figure 3:**
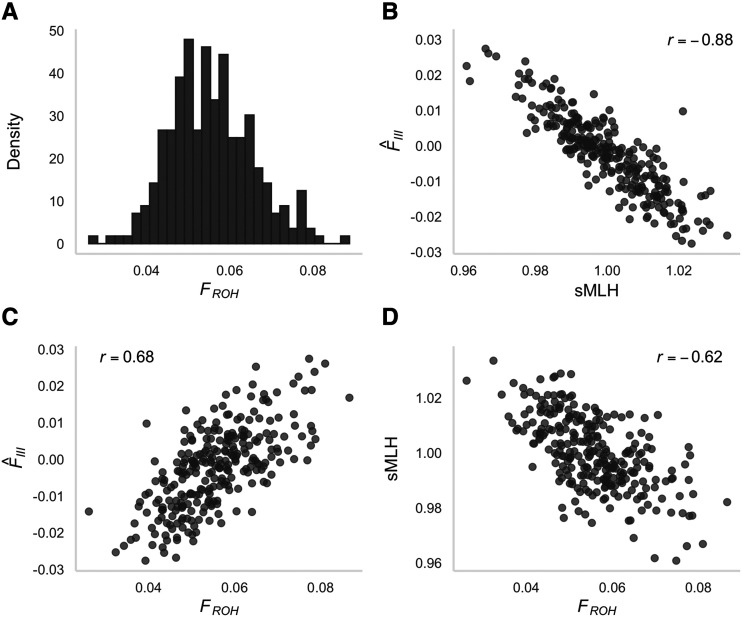
(A) Distribution of *F*_ROH_ values (the estimated proportion of the genome in ROH) for 270 Antarctic fur seals genotyped at 73,979 SNPs; (B–D) Pairwise correlations between the genomic inbreeding coefficients sMLH, ${\hat{F}}_{III}\;$and *F*_ROH_. See the Materials and methods for further details.

**Figure 4:**
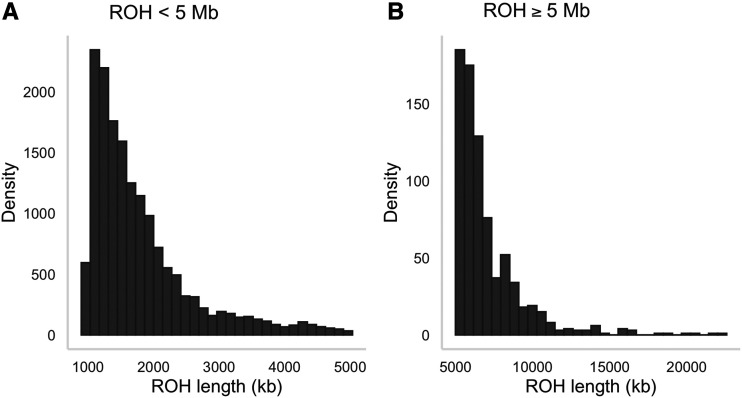
Length distributions of ROH in 270 Antarctic fur seals genotyped at 73,979 SNPs. (A) ROH segments shorter than 5 Mb are due to more recent inbreeding; and (B) ROH segments longer than or equal to 5 Mb are due to inbreeding in the more distant past.

Inbreeding was investigated using two complementary approaches. First, we quantified identity disequilibrium using the measure *g*_2_, which differed significantly from zero (0.00012, bootstrap 95% confidence interval = 0.000099–0.000150, *P* = 0.001). Second, we calculated for each individual (i) sMLH, an estimate of genome-wide heterozygosity; (ii) ${\hat{F}}_{III}$, a genomic inbreeding estimator based on the correlation of uniting gametes; and (iii) *F*_ROH_, an estimate of the proportion of the genome in ROH. *F*_ROH_ was non-zero for every individual (Figure 3A, mean = 0.06, range = 0.03–0.09) and all three genomic inbreeding measures were intercorrelated (*r* = 0.62–0.88, Figure 3B–D). ROH lengths ranged from one to 22 Mb (Figure 4), with short ROH (< 5 Mb) making up a larger proportion of the genome than medium or long ROH (≥ 5 Mb). In particular, ROH < 5Mb had a total median length of 106 Mb while long ROH ≥ 5Mb had a total median length of 19.1 Mb. ROH longer than 20 Mb were only observed in four individuals.

### Relatedness structure

**Figure 5:**
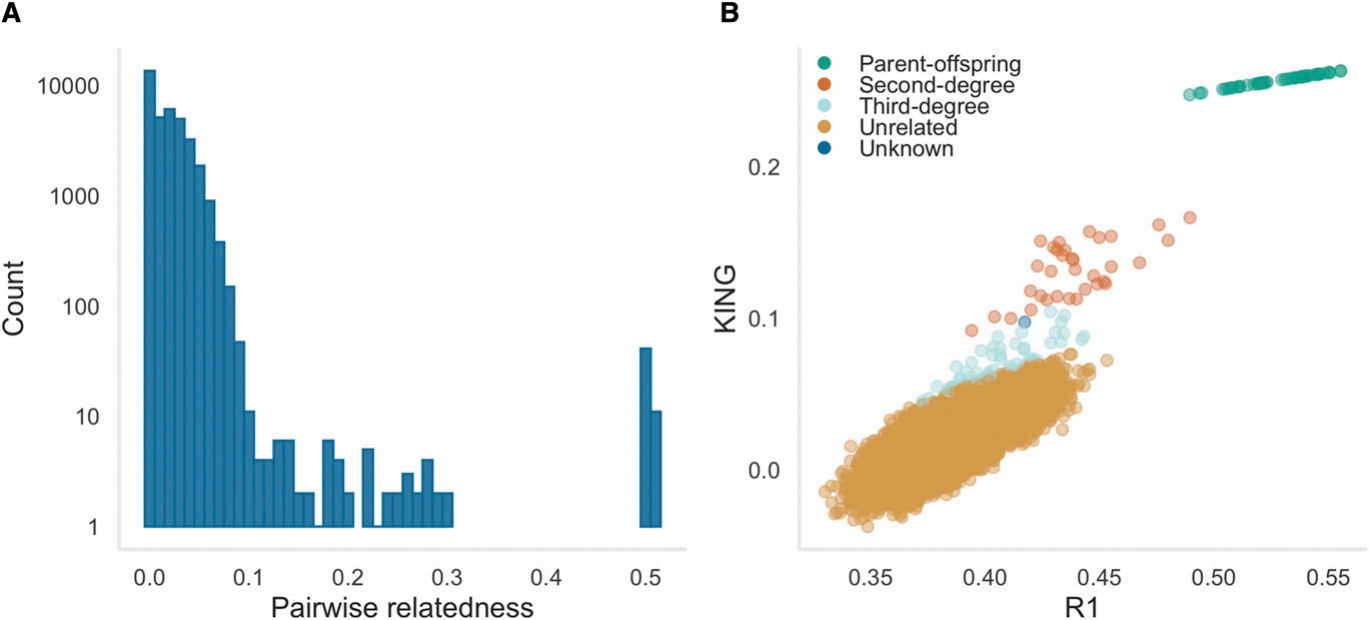
(A) Distribution of genomic relatedness values (PI_HAT) among all possible pairwise comparisons of Antarctic fur seal individuals in our dataset. Relatedness was quantified as the proportion of the genome identical by descent (IBD) between each pair of individuals based on a dataset of 6,575 maximally informative SNPs (see Materials and methods for details); (B) R1 coefficients plotted against KING-robust kinship coefficients for all individual pairwise comparisons. Points are colored according to the relationship categories inferred by comparing PLINK Z scores with the inference criteria derived in Manichaikul *et al.* (2010) and provided in Table S2.

In order to infer patterns of relatedness within our dataset, we analyzed a maximally informative dataset of 6,575 polymorphic SNPs genotyped in 270 individuals. A peak of genome-wide relatedness (PI_HAT) was present at around 0.5, corresponding to 52 mother-offspring pairs (Figure 5A). These comprised 49 pairs correctly identified based on field records, plus three additional mother-offspring pairs that were not previously known to be filial pairs. We also identified four pairs of animals that had been incorrectly assigned as biological mother-offspring pairs in our dataset. These had relatedness values of between zero and 0.24 as opposed to the expectation of around 0.5. The majority of pairwise comparisons between animals yielded genomic relatedness coefficients close to zero, although there were also a number of intermediate values, consistent with the presence of close relatives in the study population.

To investigate further, we calculated R0, R1 and KING-robust kinship values for each pair of individuals and visualized the results by plotting R1 against KING-robust kinship (Figure 5B) and R1 against R0 (Figure S3). For each pairwise comparison, we inferred a relatedness category by calculating PLINK Z scores and comparing these with the inference criteria derived in Manichaikul *et al.* (2010). Mother-offspring pairs were clearly identifiable as a cluster in the top right (Figure 5B and Figure S3A) and bottom right (Figure S3B) corners of the scatterplots and were also correctly assigned based on their PLINK Z scores. Second-degree relationships were assigned to 34 pairs of individuals that clustered together in the middle of the plots, displaying minimal overlap with other relatedness classes. When difficult to call relationships were removed (*i.e.*, those falling within 0.01 of the inference thresholds), 25 second-degree relatives remained (Figure S4A and S4B). Third-degree relationships were assigned to 63 pairs of individuals. However, while around 30 of these clustered apart from other relatedness categories in the scatterplots, the remainder were close to the kinship coefficient threshold for unrelated individuals. After removing difficult to call relationships, 24 third-degree relatives remained (Figure S4A and S4B). The majority of individual comparisons were classified as being unrelated (99.6%) and formed a large cluster in the bottom left (Figure 5B and Figure S3A) and top left (Figure S3B) corners of the plots. Full siblings were notably absent from the dataset and one pair of individuals could not be assigned to a relatedness class.

To delve into more detail, we used the pedigree reconstruction package sequoia to assign kinship categories based on a combination of known relationships and genomic data. Sequoia identified the same 52 mother-offspring pairs as described above. Of the 25 pairs of individuals assigned as second-degree relatives after difficult to call relationships were removed, 17 were similarly classified by sequoia, of which four were assigned paternal half-sib status. Three of these pairs comprised two pups born to different females in successive years, whereas the fourth pair comprised a pup and a breeding female of unknown age that were sampled seven years apart.

### Cross-species amplification

Finally, we investigated the cross-amplification potential of the array by genotyping twelve additional samples belonging to three different pinniped species. All four gray seal samples failed to pass the quality control step and were not considered further. For the Galápagos and Steller’s sea lions, the mean number of SNPs successfully called across individuals was 73,922 (range = 73,109‒74,611) and 74,130 (range= 73,164‒74,583) respectively. This is equivalent to a call rate of 96.2% for the Galápagos sea lion and 96.5% for the Steller’s sea lion. Of those SNPs that could be genotyped, 4,480 (6.1%) were polymorphic in the Galápagos sea lion and 4,191 (5.7%) were polymorphic in the Steller’s sea lion.

## Discussion

We developed a custom 85K SNP array for the Antarctic fur seal. Our efforts to prioritize high quality SNPs for tiling on the array resulted in a relatively high conversion rate, with 88.5% of the tiled loci generating readily interpretable and polymorphic genotypes. Furthermore, call rates were in excess of 99% for the majority of individuals and the genotyping error rate was low at 0.004 per reaction. Analysis of data from 270 fur seals genotyped at 75,601 polymorphic SNPs provided new insights into inbreeding and the relatedness structure of the population. Although our dataset of individuals is modest, this study provides a first impression of the promise of the array for population genomic studies of an emerging model marine mammal species.

### Design and performance of the array

Designing SNP arrays for non-model species is non-trivial and conversion rates are not always as high as desired (Helyar *et al.* 2011; Chancerel *et al.* 2011). We therefore used a suite of approaches to maximize the representation of suitable SNPs on our array. Among the most important of these were (i) using multiple callers in our transcriptome variant discovery pipeline to identify a consensus SNP panel; (ii) mapping the flanking sequences of all SNPs to the fur seal reference genome to identify loci with the most suitable genomic contexts; and (iii) using Affymetrix design scores to filter out SNPs with unfavorable flanking sequence characteristics such as high GC content and non-specific hybridization probabilities.

Overall, the comparably high conversion rate of our array suggests that these measures were successful. However, the total number of available SNPs was quite small in relation to the size of the target array, meaning that we did not have a sufficient number of SNPs in our highest priority category to fill the entire array. Consequently, careful consideration was required when establishing additional prioritization categories in order to strike a balance between SNP quantity and quality. In practice, we compromised on two main aspects. First, although we would have preferred only to tile loci with Affymetrix recommendations of “recommended”, this was not possible. Consequently, 37.9% of tiled SNPs had “neutral” Affymetrix recommendations. Second, Humble *et al.* (2018) found that loci mapping to more than one location in the reference genome were significantly less likely to convert, suggesting that probe sequence uniqueness may be an important factor to consider in SNP development. For this reason, we prioritized SNPs that mapped uniquely to the reference genome, although again we were constrained to include a number of SNPs whose flanking sequences revealed homology to more than one genomic region. As anticipated, conversion rates varied from a maximum of 93.7% for priority one SNPs down to a minimum of 66.3% for priority four SNPs. In particular, we found that SNPs that had been recommended by Affymetrix, that mapped uniquely and completely to the reference genome and that originated from RAD loci were the most likely to convert.

Another strategy that we adopted to maximize genotyping success was to include SNPs that had been pre-validated using other technologies, including Illumina GoldenGate assays (Hoffman *et al.* 2012), KASP assays (Hoffman *et al.* 2013a) and Sanger sequencing (Humble *et al.* 2018). This approach was recommended by Kim *et al.* (2018), who reported higher conversion rates on a 500K Affymetrix array for SNPs that had already been successfully genotyped on a 10K Illumina array. Unexpectedly, we found the opposite pattern, with pre-validated SNPs tending to perform worse on average than non-validated SNPs. The reasons for this remain unclear, although genotyping success was particularly low for SNPs derived from the Illumina Canine HD BeadChip. Our results therefore suggest that validating SNPs in advance may not always lead to better genotyping outcomes, especially when transferring loci from one technology to another.

As an alternative measure of genotyping success, we considered the proportion of samples that produced high quality genotypes. Only one fur seal sample out of 276 failed to pass quality control and three additional samples were considered to have failed because they fell a little short of the call rate threshold of 0.97. These numbers compare favorably with similar studies of non-model organisms (*e.g.*, Lundregan *et al.* 2018; Kim *et al.* 2018; Judkins *et al.* 2020). Overall, no relationship was found between the call rate per sample and DNA concentration, in contrast to Hagen *et al.* (2013) who reported that failed samples had significantly lower DNA concentrations than successful ones. However, all of our samples met or exceeded the recommended minimum total amount of DNA (200 ng). Consequently, our findings are in agreement with Kim *et al.* (2018), who experienced increased failure rates among samples that did not contain the recommended amount of DNA, but who found that DNA concentration did not influence genotyping success when sufficient amounts of DNA were provided.

### Levels of polymorphism

A very high proportion (97.3%) of the SNPs that successfully converted on the array were polymorphic in the Antarctic fur seal. Moreover, the true rate of polymorphism is probably higher, as several hundred SNPs were included on the array that showed *in silico* polymorphism in populations other than South Georgia, yet animals from these other localities were not genotyped on the array. Consequently, an unknown fraction of the SNPs that we have classified as monomorphic may in fact carry alleles that are private to other populations. Our main reason for including these loci was to minimize ascertainment bias in future studies that might wish to genotype animals from different locations. Indeed, studies with similar discovery schemes have demonstrated negligible ascertainment bias toward populations from which the SNPs were initially discovered (van Bers *et al.* 2012; Malenfant *et al.* 2015; Kim *et al.* 2018).

Ascertainment bias cannot be avoided with SNP arrays because high frequency polymorphisms will always be easier to discover and can be called with greater confidence due to the minor allele being present in more individuals. Nevertheless, the strong positive association that we observed between the *in silico* and empirical MAF values of seals genotyped on the array suggests that, at least for moderately variable loci, the array provides a reasonable reflection of the SFS. This in turn suggests that the discovery pool of individuals in the original RAD sequencing study was large enough to estimate MAF reasonably well for the majority of SNPs that we built into the array. In line with this, a much weaker association was observed for the transcriptomic SNPs, which were discovered by sequencing many fewer individuals. Consequently, we do not recommend the array for approaches that may be sensitive to deviations from the true SFS, such as demographic inference. Nonetheless, for most purposes, SNPs with high MAFs are beneficial as they afford greater power for a multitude of applications ranging from parentage and relatedness analysis through linkage mapping to genome-wide association studies. Consequently, we believe this array will open up a wealth of new possibilities for delving into the population genomics of this important Antarctic predator.

### Inbreeding

To assess the levels of inbreeding in our study population, we calculated three genomic inbreeding estimators (sMLH, ${\hat{F}}_{III}$ and *F*_ROH_). The resulting values were strongly intercorrelated, with *r* values ranging from 0.62 to 0.88, although associations involving *F*_ROH_ tended to be somewhat weaker. When using incomplete marker information from a SNP chip, short ROH arising from inbreeding in the very distant past cannot be reliably detected due to inadequate SNP densities (Kardos *et al.* 2016). To account for this, we only called ROH segments above a stringent length threshold. Furthermore, to avoid spurious ROH calls caused by low marker densities, we only considered ROH segments present in regions of the genome represented by high marker densities. Therefore, while our measures of sMLH and ${\hat{F}}_{\text{III}}$ have captured variation in inbreeding due to IBD segments arising from both recent and distant ancestors, our measures of *F*_ROH_ are unlikely to have captured variation in inbreeding due to very distant ancestors. Additionally, we hope to be able to further refine our estimates of inbreeding in future studies by improving the contiguity of the fur seal reference genome and by calibrating array-based measures of inbreeding by reference to whole genome resequencing data.

Nevertheless, the fact that *F*_ROH_ was non-zero in all of our samples despite the conservative nature of our analysis provides support for the presence of inbreeding in the study population. Most individuals carried ROH segments making up around 6% of the genome, with *F*_ROH_ ranging from as little as 2% in one individual to as much as 8% in four individuals. These numbers are comparable with estimates for other wild mammal populations such as the Iberian ibex (Grossen *et al.* 2018), Dryas monkey (van der Valk *et al.* 2020) and Icelandic horse (Schurink *et al.* 2019), and suggest that previously documented correlations between heterozygosity and fitness (Hoffman *et al.* 2004, 2007; Forcada and Hoffman 2014) may be due to inbreeding depression. Furthermore, the vast majority of ROH segments were shorter than 5 Mb, with only four individuals harboring ROH longer than 20 Mb. Consequently, most of the IBD observed in our study population has probably arisen from inbreeding between ancestors in the distant past, as opposed to inbreeding in more recent generations. These findings suggest that the population of Antarctic fur seals may be large enough to minimize very close inbreeding and / or that female mate choice (Hoffman *et al.* 2007) is effective at minimizing matings between close relatives. Alternatively, increasingly strong selection against highly inbred individuals over the past three decades could have resulted in the pattern we see in our data. To investigate this further, we would need to analyze a larger number of samples going back to the 1980s when the environment was more favorable, the population was stable, and inbred individuals may have been more common (Forcada and Hoffman 2014).

### Relatedness

Our study also illustrates the potential for high density SNP genotype data to recover known relationships and to uncover the relatedness structure of a sample of individuals. Genome-wide measures of relatedness based on allele sharing identified the presence of known mother-offspring pairs in our dataset. Nevertheless, we found that field-based assignments of mothers to pups are not always correct, in support of a previous study that found evidence for fostering and milk-stealing in the study colony (Hoffman and Amos 2005). Notably, full siblings were conspicuously absent from our dataset, in contrast to gray seals where around 30% of offspring are full siblings due to partner fidelity (Amos *et al.* 1995). However, mate fidelity is unlikely to be very important in Antarctic fur seals because the majority of territorial males only come ashore for a single season (Hoffman *et al.* 2003), leaving little scope for enduring sexual relationships.

We were initially surprised to discover over 90 pairs of relatives in our sample. Investigating this in greater detail, we uncovered evidence in support of the presence of a mixture of second-degree (which could potentially include half siblings, avuncular and grandparent-grandchild relationships) and third-degree relationships (such as possible first cousins). However, the classification of relatedness categories based on theoretical criteria is challenging due to variation in IBD sharing between relatives with the same pedigree (Hill and Weir 2011). This uncertainty was apparent in both the spread of data points within each relatedness category and in the degree of overlap between categories, particularly for pairs of individuals assigned as third-degree relatives. By contrast, the assignment of second-degree relationships appeared to be relatively robust, with data points displaying minimal overlap with other relatedness classes. Furthermore, of the assigned second-degree relatives, sequoia confidently identified four pairs of paternal half siblings, which we would expect to be present in the study colony given the polygynous mating system of this species (Hoffman *et al.* 2003). An additional 17 second-degree relationships were also flagged by sequoia. However, the exact nature of these relationships could not be distinguished due to most of the individuals involved having no parents assigned.

To shed further light on the relatedness structure of the study colony would require the construction of a multigenerational pedigree. In the past, we have considered this problematic due to the long generation time of the species relative to the duration of our study and the fact that not all pups are sampled every year. However, the potential for augmenting classical microsatellite-based parentage analysis with genomic information gives us new grounds for optimism.

### Cross-species amplification

Finally, we explored the cross-species amplification potential of our array by genotyping a handful of gray seals, Galápagos sea lions and Steller’s sea lions. Although none of the gray seals passed quality control, over 70,000 loci cross-amplified in both otariid species and over five percent of these were polymorphic, yielding over 4,000 polymorphic SNPs per species. This is in line with expectations set out in Miller *et al.* (2015) and demonstrates the applicability of the array for genotyping closely related pinniped species. It may also be worth considering testing the array on even less evolutionarily divergent pinniped species, most obviously other fur seal species belonging to the genus *Arctocephalus*, some of which diverged from *A. gazella* as recently as around one million years ago (Higdon *et al.* 2007), and where rates of polymorphism are expected to be as high as 20–90% (Miller *et al.* 2012).

### Conclusions

SNP arrays provide a straightforward and effective solution for generating large genetic datasets encompassing many individuals. As such, they have been instrumental in opening up a wide variety of questions to investigation in natural populations, from population genomics to quantitative genetics. This manuscript describes the successful development and implementation of a SNP array for a model marine mammal species, the Antarctic fur seal. By employing strict filtering approaches incorporating knowledge of the genomic context of each SNP, we were able to achieve comparably high rates of conversion and polymorphism. We also confirmed and built upon the results of previous studies by quantifying both inbreeding and genomic relatedness. We hope not only that our array will open up new avenues in fur seal research, but also that the protocols we developed to improve genotyping outcomes will be applicable to the design of arrays for other species.
